# Continuous negative-to-positive tuning of thermal expansion achieved by controlled gas sorption in porous coordination frameworks

**DOI:** 10.1038/s41467-018-06850-6

**Published:** 2018-11-19

**Authors:** Josie E. Auckett, Arnold A. Barkhordarian, Stephen H. Ogilvie, Samuel G. Duyker, Hubert Chevreau, Vanessa K. Peterson, Cameron J. Kepert

**Affiliations:** 1 Australian Centre for Neutron Scattering, Australian Nuclear Science and Technology Organisation, Lucas Heights, NSW 2234 Australia; 2 School of Chemistry, The University of Sydney, Sydney, NSW 2006 Australia

## Abstract

Control of the thermomechanical properties of functional materials is of great fundamental and technological significance, with the achievement of zero or negative thermal expansion behavior being a key goal for various applications. A dynamic, reversible mode of control is demonstrated for the first time in two Prussian blue derivative frameworks whose coefficients of thermal expansion are tuned continuously from negative to positive values by varying the concentration of adsorbed CO_2_. A simple empirical model that captures site-specific guest contributions to the framework expansion is derived, and displays excellent agreement with the observed lattice behaviour.

## Abstract (web summary)

Achieving control over the thermomechanical properties of functional materials is desirable, yet remains highly challenging. Here, the authors demonstrate continuous negative-to-positive tuning of thermal expansion in two Prussian blue analogues, by varying the concentration of adsorbed CO_2_.

## Introduction

Coordination frameworks have attracted sustained research interest in recent years due to their various properties of potential industrial and technological relevance^1,2^. Among the most notable of these is an ability to selectively adsorb and release small-molecule guests over many operating cycles^3–6^. This functionality is critical to the ongoing quest for efficient gas storage and separation materials in numerous industrial contexts, such as post-combustion carbon dioxide capture and hydrogen fuel storage^7–9^. The inherent porosity and relative structural flexibility that facilitate guest sorption in many frameworks also contribute to the remarkable thermomechanical behaviours exhibited by some of these materials;^10,11^ for example, dynamic motions of framework structural components into the available void spaces give rise to negative thermal expansion (NTE) in a number of frameworks^12–16^. NTE and zero thermal expansion (ZTE) are considered desirable material properties for a variety of applications where the minimisation of strain during temperature cycling is important, and the ability to tune the rate of expansion of a device component to compensate for the positive expansion of another component is particularly advantageous. As the inclusion of guests or other modifiers in the voids of porous frameworks can directly influence the dynamic motions associated with NTE, the interplay between guest concentration and temperature introduces considerable complexity to the study of thermomechanical properties in host–guest framework systems^17–19^.

**Fig. 1:**
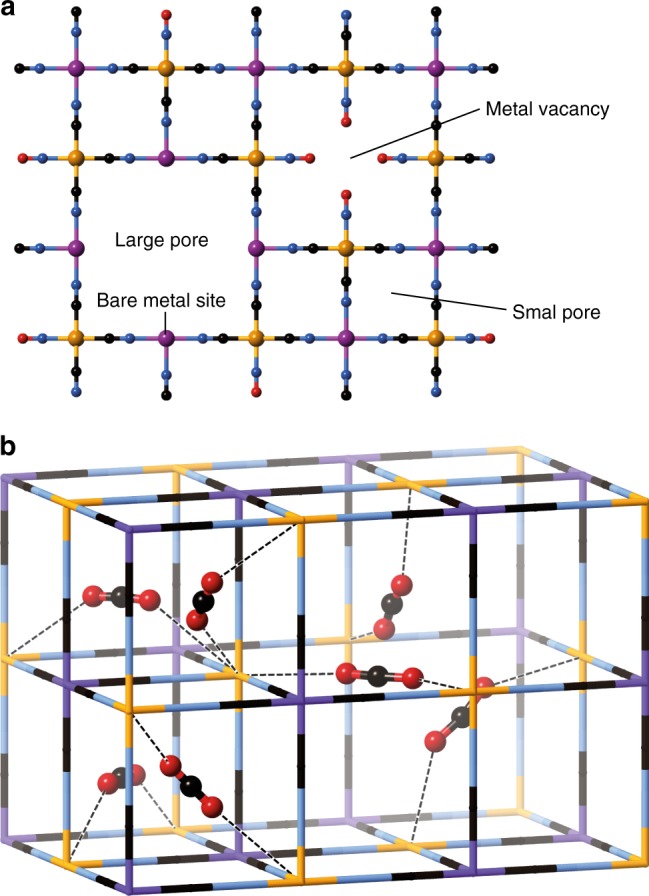
Visual representations of Prussian blue-type frameworks. **a** Network of a defective TMNP framework illustrating the different pore types. The large pore arises from vacancies of the Fe(CN)_5_NO unit in TMNPs or of the M′(CN)_6_ unit in PBAs. The metal vacancy pore arises from the presence of non-bridging NO ligands in TMNPs and does not occur in hexacyanidometallate PBAs. Note that, while they are shown as separate here, the vacancy pores may border upon or overlap with others of the same or different type. **b** Fragment of the FeCo-PB structure containing its theoretical maximum CO_2_ content of one “Site A” CO_2_ molecule in each of the four small pores and three “Site B” molecules in the large pore. Dashed lines indicate interactions between CO_2_ and the Fe sites. The host and guest atomic positions were previously determined by NPD^37^. Colours: Fe (orange); Co (purple); C (black); N (blue); O (red)

The Prussian blue family (*M*_*y*_[*M*′(CN)_6_], *M* and *M*′ = transition metals) comprises one of the simplest coordination framework structure types and is a model system often suited to the study of phenomena common to more complicated systems. The archetypical Prussian blue framework is constructed of cyanido (CN^−^) ligands bridging the octahedrally coordinated *M* and *M*′ ions, which are arranged in a “rock salt” ordering pattern on a cubic lattice. *M* and *M*′ may be chosen with various combinations of valence states^20–24^, generating a series of structurally analogous frameworks known as Prussian blue analogues (PBAs). Many of the possible valence combinations result in well-defined concentrations of randomly distributed hexacyanidometallate vacancies in order to preserve charge balance (Fig. 1). The faces of the cubic “large pore” created by an absent hexacyanidometallate unit each contain a bare metal site that can act as an attractive site for guest binding^20,25^. An important PBA variant is the transition metal nitroprusside (TMNP) family^26^, in which [Fe(CN)_5_NO] is substituted for [*M′*(CN)_6_] and additional “metal vacancy pores” are introduced adjacent to every six non-bridging NO ligands.

Various PBA and derivative frameworks have been shown to exhibit isotropic NTE over a wide temperature range due to a mechanism in which transverse ligand vibrations into the adjacent void spaces cause the metal-centred octahedral units to be drawn closer together^17,21^. Previous studies have demonstrated that the coefficient of thermal expansion (CTE, *α*) of PBA and other NTE-active frameworks can be varied considerably, even from negative to positive values, by the substitution of different metal ions^21,27,28^. It has also been shown that NTE behaviour in certain frameworks is converted to positive thermal expansion when solvent molecules are present in the pores^17,29,30^ and that intermediate CTE values can be achieved by partial desolvation at high temperatures^18^. Substitution of different solvent guests has also been shown to modify CTEs in uniaxial and biaxial NTE materials^31,32^. The presence of extra-framework ionic species has additionally been shown to influence thermal expansion behaviour^33,34^. However, to our knowledge, no studies have yet shown how the CTE of one framework can be tuned continuously and reversibly over a wide range of positive and negative values using a dynamic stimulus. Here we demonstrate a new and facile method of negative-to-positive CTE tuning by altering the concentration of CO_2_ guest molecules adsorbed in the pores, as exhibited by the TMNP Co[Fe(CN)_5_NO] (hereafter Co-NP) and the PBA Fe_3_[Co(CN)_6_]_2_ (hereafter FeCo-PB). A simple empirical model for the modifying effects of guest concentration on the thermal expansion characteristics of the empty framework is also developed, allowing the observed behaviour to be understood in relation to the guest-binding locations and host–guest interactions within the host frameworks.

## Results

### Tuneable guest-dependent thermal expansion

**Fig. 2:**
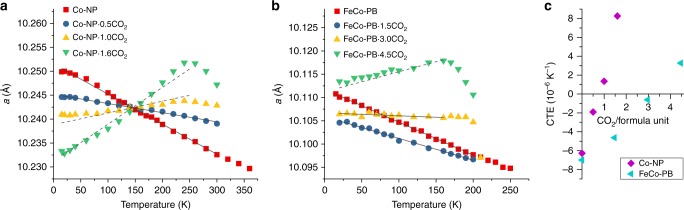
CO_2_-dependent thermal expansion trends for Co-NP and FeCo-PB. **a**, **b** Symbols represent the experimentally determined cubic lattice parameters of Co-NP and FeCo-PB at various CO_2_ loadings and temperatures. Error bars are smaller than the symbols. Solid lines represent linear regression fits over limited data ranges (see Table 1); these fits were used to parameterise the formulae derived in the text (see Eqs. (1) and (2)). The dashed trend lines are predicted by the parameterised model. **c** Evolution of the CTE values obtained by linear regression fits against all data series

The unit cell parameters of Co-NP and FeCo-PB at various CO_2_ loadings were extracted by Le Bail analysis of variable-temperature neutron powder diffraction (NPD) data collected upon warming (Fig. 2a, b; experiment details in ESI). The sharp downturn observed at high temperatures for the highest CO_2_ concentrations is attributed to the onset of thermally induced CO_2_ desorption, which causes the lattice parameter to approach that of the guest-free framework. For both FeCo-PB and Co-NP, linear CTEs (*α*_*a*_ = (1/*a*)(d*a*/d*T*)) calculated from linear regression fits in the low-temperature range display a continuous increase with increasing CO_2_ concentration, from strongly negative for the empty framework to positive for the highest CO_2_ concentrations measured (Table 1). The values obtained for the empty frameworks are typical of guest-free PBAs^21^.

**Table 1:** Coefficients of thermal expansion obtained by linear regression fits to refined lattice parameters

*x* (CO_2_ guests per formula unit)			*α*_*a*_ (×10^−6^ K^−1^)	Fitted range (K)
Co-NP				
0			−6.12(8)	15–300
0.5			−1.87(5)	15–300
1.0			+1.32(8)	15–240
1.6			+8.1(3)	15–240
FeCo-PB	Site A	Site B		
0	0	0	−6.99(6)	15–200
1.5	1.5	0	−4.63(10)	20–200
3.0	2.0	1.0	−0.6(2)	20–160
4.5	2.0	2.5	+3.30(17)	20–160

The tunability of thermal expansion in these frameworks is rationalised by considering the nature of low-energy, rigid-unit NTE modes operating in PBAs and their derivatives^13,14^. As the empty pore space of the framework becomes filled with guests, the transverse vibrations of the bridging cyanides, which ordinarily lead to concerted, volume-reducing distortions of the framework, are increasingly impeded by steric interactions with the guest molecules. This explanation has also been proposed for the suppression of NTE by the presence of solvent molecules in the *M*[Pt(CN)_6_] (*M* = Zn, Cd)^17^ and Cd(CN)_2_^18^ frameworks.

### Simple expansivity model

In Co-NP, the intersection of all lattice parameter trends at a single point is suggestive of competition between two linear factors of opposite sign affecting thermal expansion simultaneously and negating each other at a certain temperature. We assign these competing effects to two different interactions known to occur between guest molecules and porous frameworks. The first has been reported in zeolites^35^ and MOFs^36^ and is characterised by contraction of the framework at low temperatures due to the attractive nature of the guest acting on multiple framework atoms; this may be termed the “static” effect. Simultaneously, the guests exert an expansive pressure on the framework with increasing thermal excitation, which may be called the “dynamic” or “thermal” effect. Contraction of the framework due to the static effect of CO_2_ is considered to be temperature independent and has the effect of decreasing the 0 K intercept of the thermal expansion trend with increasing guest concentration. By contrast, the temperature-dependent dynamic effect positively increases the rate of thermal expansion with increasing guest concentration. The following simple expression captures the static and dynamic effects on thermal expansion behaviour:

1 $$a = a_0 + Sx + \left( {m_0 + Dx} \right)T$$

where *x* is the guest concentration, *T* is temperature, *a*_0_ is the extrapolated lattice parameter of the empty framework at 0 K, *m*_0_ is the thermal expansion gradient of the empty framework, *S* is the static parameter or contractive guest effect (Å), and *D* is the dynamic thermal parameter or expansive guest effect (Å K^−1^). It may be seen that when *DT* = *−S*, the concentration-dependent expansive and contractive effects are cancelled out; consequently, thermal expansion trends for all guest concentrations intersect at *T* = *−S/D*. Furthermore, zero thermal expansion (ZTE) behaviour is predicted when the guest concentration *x* = −*m*_0_/*D*. In the case of Co-NP, the calculated crossover temperature for all curves is 145 K and ZTE is predicted to occur at a guest concentration of 0.72(2) CO_2_/formula unit, with the associated unit cell parameter *a* = 10.2424(3) Å. The values determined for *S* and *D* are reported in Table 2; details of their calculation are given in Supplementary Information. Thermal expansion trends predicted by the model are represented with dashed lines in Fig. 2a, revealing excellent agreement with the experimental data below the onset temperature of CO_2_ desorption.

**Table 2:** Parameter values determined for the prediction of guest-dependent thermal expansion trends in Co-NP and FeCo-PB

Co-NP (using Eq. (1))		FeCo-PB (using Eq. (2))	
*a*_0_	10.25150(13) Å	*a*_0_	10.11177(7) Å
*m*_0_	−6.28(8) × 10^−5^ Å K^−1^	*m*_0_	−7.07(6) × 10^−5^ Å K^−1^
*S*	−12.7(3) × 10^−3^ Å	*S*_*A*_	−4.00(9) × 10^−3^ Å
*D*	8.74(19) × 10^−5^ Å K^−1^	*D*_*A*_	1.63(8) × 10^−5^ Å K^−1^
		*S*_*B*_	3.0(3) × 10^−3^ Å
		*D*_*B*_	3.3(3) × 10^−5^ Å K^−1^

*a*_0_ and *m*_0_ originate from linear regression fits to the empty framework data

### Multi-site expansivity model

The wider applicability of this approach for modelling thermal expansion in cubic systems is expected to be dependent upon the nature of guest-binding sites within the host framework. Indeed, attempts to model the thermal expansion behaviour of FeCo-PB using Eq. (1) were not successful. Ogilvie et al.^37^ previously identified two distinct “bridged” binding sites for CO_2_ in FeCo-PB at 15 K: Site A in the large pore (maximum capacity 2 CO_2_ molecules per formula unit), and Site B located over the small-pore squares. A modified equation taking into account different static and dynamic contributions of guests bound at Sites A and B takes the form

2 $$a = a_0 + S_Ax_A + S_Bx_B + \left( {m_0 + D_Ax_A + D_Bx_B} \right)T$$

where the subscripts *A* and *B* denote guest concentrations and thermal expansion parameters corresponding to CO_2_-binding sites A and B, respectively. Assuming maximal occupation of Site A followed by Site B up to the nominal guest concentration (Table 1), a surprisingly good approximation of the experimental data at 4.5 CO_2_ is obtained using the extended model in Eq. (2), lending support to the suitability and predictive power of this model for multisite sorbents. The fact that the model succeeds by assuming perfectly sequential filling of Sites A and B also suggests that thermal randomisation of CO_2_ guests over both sites is negligible for all measured CO_2_ concentrations up to the desorption onset temperature; this is consistent with the highly optimal binding geometry identified for Site A^37^, which is expected to give it a significant energetic advantage over Site B.

The *D*_*A*_ and *D*_*B*_ values in Table 2 indicate that CO_2_ guests bound at Site B in FeCo-PB cause a more rapid positive increase of the CTE than those at Site A. Given that Site B is positioned closer than Site A to the CN^−^ ligands^37^, this observation is consistent with the suppression of NTE being caused by interference of CO_2_ guests with the CN^−^ vibrational modes. Guests at Site B are also found to exert an expansive static effect on the FeCo-PB framework (*S*_*B*_ > 0), probably reflecting the weaker host–guest interaction at B that is associated with the strong guest preference for Site A. By contrast, the fact that Co-NP was satisfactorily modelled using the simpler Eq. (1) suggests that it contains only one unique CO_2_-binding site or multiple sites of very similar character. This prediction is borne out by the preliminary results of Rietveld refinements against the NPD data for Co-NP, which have only identified a single, highly disordered CO_2_-binding site located at the centre of the small pore (see Supplementary Fig. 2). A detailed analysis of these refinements will be presented in a future publication.

### Summary

Variable-temperature neutron diffraction data reveal for the first time that the CTEs of cyanide-based frameworks can be varied from negative to positive values by progressive adsorption of guest molecules into the pores. The evolution of CTE with guest concentration can be modelled on the basis of two independent contributions from each guest-binding site, as demonstrated by applying one-site and two-site versions of the model to Co-NP and FeCo-PB, respectively; this result further highlights the value of identifying and characterising individual guest-binding sites and understanding host–guest interactions in sorbent systems. The generally linear relationship between CTE and CO_2_ concentration offers a method of control for the tuning of thermal expansion properties in porous solids that is more facile than framework compositional changes or ex situ removal of solvent molecules, allowing the target concentration for generating ZTE behaviour to be calculated easily from the model equations and conveniently achieved through controlled gas dosing. This finding opens new opportunities for tailored thermal expansion properties to be dynamically engineered in porous framework materials over low-to-moderate temperature ranges.

## Methods

### Synthesis of Fe_3_[Co(CN)_6_]_2_ (FeCo-PB)

Aqueous solutions of FeCl_2_·H_2_O (2.13 g, 10.7 mmol) and K_3_Co(CN)_6_ (1.69 g, 5.08 mmol) were mixed dropwise with vigorous stirring. The solution was allowed to stand for 24 h before the precipitate was filtered and washed with water (100 mL), followed by ethanol (100 mL), and dried in air to yield Fe_3_[Co(CN)_6_]_2_·*x*H_2_O as a pale yellow powder (1.76 g; *x* ≈ 10; yield ~89%). Desolvation was achieved by grinding the sample to a fine powder and heating it in a glass sample tube at 423 K under high vacuum (~10^−5^ mbar).

### Synthesis of Co[Fe(CN)_5_NO]·4H_2_O (Co-NP)

A solution of Na_2_[Fe^II^(CN)_5_NO]·2H_2_O (1.000 g, 3.36 mmol) in 200 mL deionised water was stirred until fully dissolved, followed by addition of Co(NO_3_)_2_·6H_2_O (0.978 g, 3.36 mmol) to the stirred solution. After continuously stirring for 16 h, the reaction mixture was centrifuged at 4000 rpm and the supernatant discarded. The product was washed four times by adding fresh deionised water, agitating by hand and then repeating the centrifuge procedure. The resulting solid was air dried for 48 h and then stored in a desiccator for 24 h. The product was a pale reddish brown powder (yield: ~70%).

### Neutron powder diffraction

Variable-temperature NPD data were collected for Co-NP using the high-intensity neutron powder diffractometer WOMBAT^38^ at the Australian Nuclear Science and Technology Organisation (ANSTO). A 2.142 g sample was loaded into a 6-mm-diameter cylindrical vanadium can inside a helium-filled glovebox and attached to a custom-designed gas-delivery centrestick equipped with in situ gas delivery capabilities, which has been described elsewhere^39^. The incident neutron wavelength (~2.41 Å) was refined against an Al_2_O_3_ standard reference material (NIST SRM 676). NPD data were collected in the angular range 17° < 2*θ* < 137° at various temperature intervals upon heating, with the sample temperature stabilised within 1 K of the set point prior to data acquisition. A Hiden Isochema IMI manometric dosing system was then used to dose the sample cumulatively with aliquots of CO_2_ gas loaded into a dosing manifold of calibrated volume, allowing the quantity of gas in each dose to be determined from the manifold pressure. All gas doses were applied to the sample at 220 K followed by slow cooling to 15 K to avoid the deposition of solid CO_2_ in the sample chamber; the attainment of near-zero pressure measured inside the sample vessel was taken to indicate complete uptake of the dose by the sample. The entire series of variable-temperature NPD measurements was repeated after each CO_2_ dose.

Data reduction was performed using LAMP^40^ and included a correction applied for the Debye–Scherrer ring curvature. Lattice parameters for the Co-NP phase were extracted using the Le Bail method as implemented in the program GSAS^41^, which was used in conjunction with the EXPGUI^42^ graphical interface. A pseudo-Voigt peak profile with axial divergence (GSAS Type II) and 16-term shifted Chebyshev background function were fitted to the data. Manual analysis was carried out for the first NPD pattern in each temperature series, followed by sequential (automated) Le Bail extractions over the rest of the series, using the final refined parameters from the previous pattern as a starting point for each subsequent extraction.

Details of the NPD experiments and data treatments for FeCo-PB were very similar to those described here for Co-NP and can be found in Supplementary Information accompanying a previous publication by Ogilvie et al.^37^. Examples of the Le Bail fit profiles obtained for FeCo-PB and Co-NP are shown in Supplementary Fig. 1 accompanying this article, and agreement indices for all fits are summarised in Supplementary Table 1.

## Electronic supplementary material

Supplementary Information

## Data Availability

The authors declare that the data supporting the findings of this study are available within the article and its Supplementary Information files. All other relevant data supporting the findings of this study are available on request.
