# Supplementary material for: Continuous negative-to-positive tuning of thermal expansion achieved by controlled gas sorption in porous coordination frameworks
Source: Nat Commun. 2018 Nov 19;9:4873. doi: 10.1038/s41467-018-06850-6 (PMC6242975; doi:10.1038/s41467-018-06850-6)
Supplement: Supplementary file 1 — Supplementary Information [file 41467_2018_6850_MOESM1_ESM.pdf]

SUPPLEMENTARY INFORMATION

Continuous negative-to-positive tuning of thermal expansion achieved by controlled gas sorption in cyanide-based frameworks

Josie E. Auckett, Arnold A. Barkhordarian, Stephen H. Ogilvie, Samuel G. Duyker, Hubert Chevreau, Vanessa K. Peterson and Cameron J. Kepert

Supplementary Notes

Further details of empirical parameter determinations for the guest-dependent thermal expansion models

The values of D and S for Co-NP were solved using Equation (1) after obtaining m_0 and a_0 (the gradient and intercept, respectively, of the empty framework NTE) and the gradient and intercept of one other data series in Figure 2(a) by linear regression. Equation (1) was then used to generate predicted thermal expansion trends for the remaining two data series (1.0CO_2 and 1.6CO_2 loadings); these predictions are indicated by dashed lines in Figure 2.

A similar procedure was followed for the solution of S_A, D_A, S_B and D_B for FeCo-PB using Equation (2), except that two pairs of regression-fitted gradient and intercept values were required in order to solve for the four arbitrary parameters, leaving only one experimental data series in Figure 2(b) to be predicted by Equation (2).

Further discussion of lattice parameter changes upon thermal desorption of CO_2

The downturns in the framework lattice parameters at higher temperatures result from the desorption of CO_2 molecules from the pores (Figure 2). For FeCo-PB·4.5CO_2, this begins at *ca*. 170 K and is assumed to correspond to desorption of CO_2 from Site B, this being the higher energy adsorption site. Notably, the desorption begins at a slightly lower temperature for the 4.5CO_2 loading relative to the 3CO_2 loading. Both of these loadings contain CO_2 at site B, and therefore this initial desorption will occur from this site, but they are not energetically equivalent as the overall energy is expected to increase (i.e., weaker guest binding) as Site B becomes increasingly populated. Desorption from the more strongly binding site A is expected to occur at a higher temperature, outside the range of our experiment.

On the other hand, Co-NP, which we suspect contains a single relatively strongly binding bare metal site, begins to desorb CO_2 at a higher temperature than FeCo-PB, *ca*. 250 K.

Supplementary Figures

a

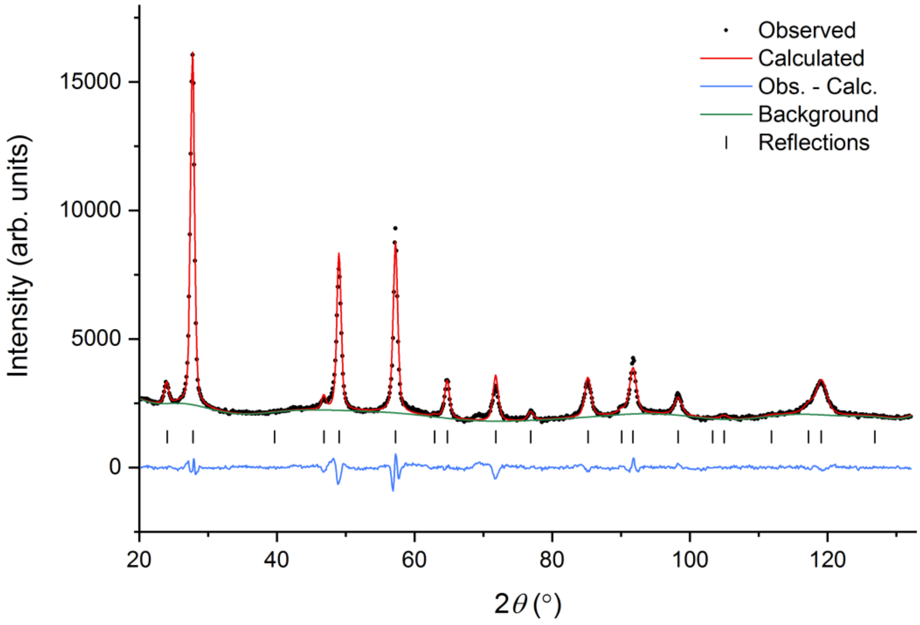

b

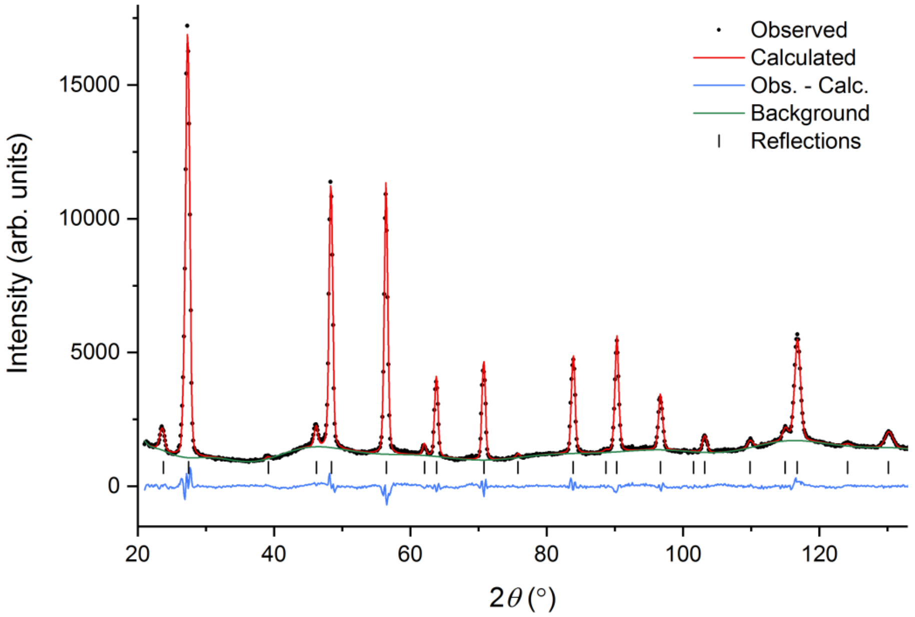

Supplementary Figure 1. Example Le Bail fits obtained against NPD data. (**a**) Empty FeCo-PB at 15 K. (**b**) Co-NP at 100 K.

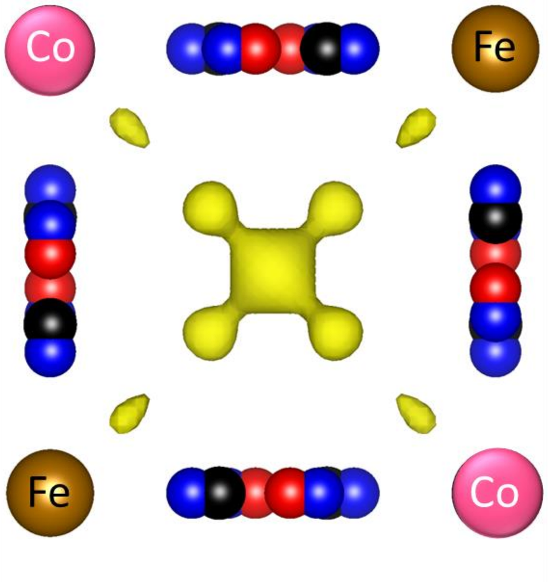

Supplementary Figure 2. Fourier difference map of CO_2 guest binding in Co-NP obtained by Rietveld refinement against NPD data. The yellow surface represents positive residual nuclear density and is interpreted as a single, disordered binding CO_2 site located at the centre of the cubic pore. The surface is tetrahedral in symmetry, with each of the rounded "corners" pointing towards a Co node. The overlapping CN and NO groups depicted in the image reflect the compositional and positional disorder of the ligands in the cubic structure model. Atom colours: Fe (brown); Co (pink); C (black); N (blue); O (red).

Supplementary Tables

Supplementary Table 1. Ranges of agreement indices obtained for sequential Le Bail fits over the NPD data series.

Sample	Guest loading	# patterns	*wRp*
FeCo-PB	empty	27	2.93–3.41%
	1.5CO_2	16	3.48–4.78%
	3.0CO_2	17	3.15–5.12%
	4.5CO_2	16	3.04–5.60%
Co-NP	empty	28	3.45–3.39%
	0.5CO_2	17	4.59–4.68%
	1.0CO_2	17	4.52–4.17%
	1.6CO_2	17	4.67–4.41%
